# Upstream region of *OprD* mutations in imipenem-resistant and imipenem-sensitive *Pseudomonas* isolates

**DOI:** 10.1186/s13568-021-01243-3

**Published:** 2021-06-05

**Authors:** Masoumeh Kiani, Akram Astani, Gilda Eslami, Mansoor Khaledi, Hamed Afkhami, Soodabeh Rostami, Mohadeseh Zarei, Nahid Rezaei Khozani, Hengameh Zandi

**Affiliations:** 1 Department of Microbiology, Faculty of Medicine, Shahid-Sadoughi University of Medical Sciences, Yazd, Iran; 2 Department of Parasitology and Mycology, School of Medicine, Shahid Sadoughi University of Medical Sciences, Yazd, Iran; 3 Department of Microbiology, Faculty of Medicine, Shahed University, Tehran, Iran; 4 Nosocomial infections Research Center, Isfahan University of Medical Sciences, Isfahan, Iran; 5 Department of Microbiology, Faculty of Medicine, Kashan University of Medical Sciences, Kashan, Iran

**Keywords:** *P. aeruginosa*, Antimicrobial resistance, *oprD*, Upstream, Imipenem, Promoter

## Abstract

The current study was aimed at investigating the prevalence of the mutations upstream of the *oprD* coding region and its promoters among imipenem-resistant and sensitive *Pseudomonas aeruginosa* isolated from educational hospitals in Yazd City, Iran. All isolates were identified by the conventional biochemical tests. Then, the antibiotic resistance of these isolates was determined using the disk diffusion method according to the CLSI guidelines. Also, the E.test was performed to determine the minimum inhibitory concentrations (MIC) of imipenem. The mutations of this gene were recognized by the amplification of this region and subsequently sequenced. Sequencing of the genomic region upstream of *oprD* these regions were done in the 29 clinical strains. Statistical analysis was done by the statistical software SPSS-18. Seventy (77.7%) of isolates had MIC ≥ 16 and were resistant to imipenem. Mutations of the upstream of the *oprD* gene and its promoters were seen in 25 (86.2%) isolates and 4 isolates had no mutation. One isolate had a base substitution A→Cat nt 25 in the coding region and this isolate had a point mutation leading to an amino acid change at positions 9 (I→L). Our study results indicated that none of the strains had mutation in Shine-Dalgarno and the point mutations were the most common mutations upstream of the *oprD* coding region among *P. aeruginosa* isolates. Mutations were observed in imipenem-resistant isolates and it seems this mechanism is effective in resistance of isolates to imipenem and this confirmed that the indiscriminate use of antibiotic should be controlled.

## Key points

- 1. Carbapenems, mainly imipenem and meropenem, are important and useful antibiotics for the treatment of infections due to multidrug-resistant *Pseudomonas.*
- 2. The Loss or mutations of outer membrane porin (*OprD*) and promoters of the *oprD* gene appears to be the most common mechanisms of intrinsic resistance to imipenem.
- 3. The antibiotic resistance of these isolates was determined using the disk diffusion method and E. test according to the CLSI guidelines. The mutations were recognized by the amplification of this region and subsequently sequenced.
- 4. All the imipenem-resistant isolates had mutations and the mutation was not seen in susceptible isolates
- 5. In Iran, there is little information about the contribution of different mechanisms to imipenem resistance in these isolates, especially about *oprD* mutations in the upstream region of gene and promoter in imipenem-resistance isolates.

## Introduction

*Pseudomonas aeruginosa* is an opportunistic pathogen that causes a variety of infections in immunocompromised patients.‏ In recent years, Antibiotic resistance of *P. aeruginosa* is increasing and the selection of suitable treatments has become difficult and is associated with increased morbidity and mortality (Riera et al. 2011; Yan et al. 2014).

Carbapenems, mainly imipenem and meropenem, are important and useful antibiotics for the treatment of infections due to multidrug-resistant Pseudomonas. Carbapenems are a class of β-lactam antibiotics with good antimicrobial activity against *P. aeruginosa *(Liste et al. 2009*; *Ocampo-Sosa et al. 2012). Carbapenem resistance of *P. aeruginosa* is mainly due to a combination of different factors, including low permeability of outer membrane porin and mutations in the gene encoding *OprD*, the production of the *AmpC* bate-lactamases, overproduction of efflux systems, and producing Carbapenemase (Hancock and Brinkman, 2002; Pirnay et al. 2002; Rostami et al. 2018). However, among these mechanisms, the Loss or mutation of outer membrane porin (*OprD*) and promoter of this gene appears to be the most common mechanisms of intrinsic resistance to imipenem and a lesser extent to meropenem. This mechanism causes blocking of the entrance of carbapenems particularly imipenem into a bacterium (Amin et al. 2005; Shen et al.^2015^).

*OprD*, an outer membrane porin is a semipermeable barrier and substrate-specific a penetrable protein consisting of 443 amino acids that allows the diffusion of sugars, small peptides, basic amino acids, and carbapenems typically imipenem into the cell (Cowan et al. 1992; Pirnay et al. 2002).

*OprD* mediated resistance occurs as a result of decreased transcriptional expression of *oprD* and imipenem resistance has been associated with (i) mutations that inactivate or destroy at least one of the *oprD* promoters, (ii) premature termination of *oprD* transcription, (iii) co-regulation with trace metal resistance mechanisms such as Zinc and copper, (iv) salicylate-mediated reduction, and (v) decreased transcriptional expression via co-regulation with the multidrug efflux pump encoded by mexEF-oprN (Amin et al. 2005).

The typed of mutations in the *oprD* gene and upstream regions and promoters of this gene are various such as nucleotide deletions, insertions, and point mutations that have been recognized to be the major mechanisms leading to inactivation of the *oprD* gene and promoter in imipenem-resistant isolates of *P.aeruginosa* (Gutiérrez et al. 2007; Pirnay et al. 2002). Transcription of *oprD* in *P. aeruginosa* PAO1 initiates with equal frequencies from two start sites, located 23 bases (SS1) and 71 bases (SS2) upstream of the structural gene. In the previous investigation, two or three types of imipenem-resistance mutants in clinical isolates were observed. The major type involves deletion and point mutations (Lynch et al. 1987). These well-known alterations are commonly reported, include point mutations or insertion sequences (ISs) inactivating in the resistance to imipenem, especially in Iran. Therefore, this study aimed to evaluate the prevalence of mutations upstream of the *oprD* coding region and its promoters in imipenem-resistant and -sensitive *Pseudomonas aeruginosa* isolated from educational hospitals.

## Materials and methods

### Bacterial isolates

In a descriptive study, 90 isolates of *P. aeruginosa* were collected from June 2018 to April 2019 at the Teaching Hospitals of Shahid Sadoghi University of Medical Science, Yazd, Iran. These isolates were originated from different clinical specimens of hospitalized patients, including blood, burn wounds, urine, lungs, etc.

### Antimicrobial susceptibility testing

After transferring the plate containing Gram-negative rod colonies to the Laboratory of Microbiology, suspected colonies were identified by Gram staining and conventional biochemical tests such as catalase, oxidase, growth in 42 °C, oxidative/fermentative test, and differential media such as TSI (Merck, Germany). Isolate identified as *P. aeruginosa* were stored at 7 °C in trypticase soy broth (Merck) supplemented with a 20 °C glycerol unit.

### Minimum inhibitory concentration and phenotypic confirmatory tests

Antibiotic susceptibility testing of the isolates was performed using the disk diffusion method (Kirby-Bauer) according to Clinical and Laboratory Standard Institute guideline (CLSI, 2019) using Mueller-Hinton agar (Merck, Germany) and Imipenem, meropenem, ertapenem, ciprofloxacin, ceftazidime, Cefepime, ceftriaxone, gentamicin, and tobramycin (MAST, UK). *P. aeruginosa* ATCC27853 was used as quality control. The Minimum Inhibitory Concentration (MIC) of imipenem was performed by E. test strips (Liofilchem, Italy) as described in the manufacturer's instructions. MIC breakpoint was defined according to CLSI guidelines (CLSI, 2019).

### DNA extraction

DNA extraction was performed using by salting out method and was stored at – 20 °C until further use (18).

### PCR for detection of oprD gene

PCR technique was performed. Primers were developed for each gene using Primer 3. The primers used for DNA amplification, as follows: 5′-AGACATGCCGTGGATACAAA0-3′ for the forward and 5′- AGTGCTACCTGCGGAAACC -3′ for the reverse primers. The final optimized PCR reaction consisted of 0.5 μl MgCl2 (100 mM), 0.5 μl dNTP (10 mM), 0.2 µl (1 unit) Taq DNA polymerase (Cinnagen, Iran), 1 µl of each primer (10 pmol) (Alpha DNA, Canada), 2.5 µl PCR buffer (10 X), and 0.5 μl of DNA template (100 μg/ml) in a total volume of 25 μl with double distilled water. DNA amplification was carried out with a thermocycler (Quanta Biotech, England), PCR amplification was performed as follows: one cycle at 95 °C for 300 s, then 30 cycles at 95 °C for 45 s, 56 °C for 45 s, and 72 °C for 60 s and a final extension at 72 °C for 10 min using an initial denaturation step for 5 min at 94 °C (one cycle), followed by 35 cycles of 1 min at 94 °C, 1 min at 50 °C, and 1 min at 72 °C. The amplified products were analyzed by 1.5% (w/w) agarose gel electrophoresis and were visualized on an ultraviolet illumination after staining with ethidium bromide.

### DNA sequencing and analyses of sequence data

According to imipenem MIC results, 29 isolates were selected randomly (due to Financial Limitations) for evaluation of the mutations. We amplified and sequenced the genomic region upstream of *oprD* genes in the imipenem-resistant (n  =  25) and imipenem-sensitive (n  =  4) bacteria. For DNA sequencing, upstream regions and fifty-four (54) primary nucleotides of the *oprD* gene were sequenced. The sequence results were aligned and analyzed using MEGA 6 software and CLUSTAL W2, Vector NTI Advance version9.0.0 software (InforMax; Invitrogen). Protein alignments were carried out using. ClustalW2 (http://www.ebi.ac.uk/Tools/msa/clustalw2/). A mutation in the promoter and the upstream coding region of the *OprD* gene (Table 3) was identified by DNA sequencing.

### Statistical analysis

The data were analyzed using the Statistical Program for Social Sciences version 18. (SPSS Version. 18 IBM, Chicago, IL, USA). For the analysis of data, chi-square tests were employed to calculate the P-value. Statistical significance and levels were set at P < 0.05.

## Results

### Bacterial isolates

Of 90 *P. aeruginosa* isolates, 38.9%, 20%, and 13.3% of them were isolated from burn wounds, urine, and wound specimens respectively. The Sources of *P. aeruginosa* isolates according to the hospital ward include Burn (43.3%), ICU (22.2%), Internal (15.6%), Surgery (11.1 %), and other wards (7.7 %).

### Antibiotic resistance patterns

The frequency of resistance to carbapenems was as follows: imipenem 48.9%, meropenem 56.6%, and Ertapenem 52.5%. The results of antimicrobial susceptibility testing using the disk diffusion method are shown in Table 1. The results of the MIC of Imipenem by an E-test method are shown, 77.7% of isolates had MIC ≥ 16 and were resistant to imipenem and 22.2% of isolates had MIC ≤4 and Sensitive to imipenem.

**Table 1:** Frequency of antibiotic resistance patterns in *P. aeruginosa* isolated from clinical sample

Antibiotic	Sensitive No. (%)	Semi sensitive No. (%)	Resistant No. (%)
Imipenem (10 μg)	44 (48.9)	2 (2.2)	44 (48.9)
Meropenem (10 μg)	38 (42.2)	1(1)	51(56.6)
Ertapenem (10 μg)	29 (32.2)	14 (15.3)	47 (52/5)
Ciprofloxacin (5 μg)	46 (51.1)	4 (4.4)	40 (44/4)
Ceftazidime (30 μg)	32 (35.6)	13 (14)	45 (50)
Cefepime (30 μg)	40 (44.4)	2 (2.2)	48 (53.3)
Ceftriaxone (30 μg)	26 (28.9)	17(18.9)	48 (53.3)
Gentamicin(10 μg)	38 (42.2)	7 (7.8)	45 (50)
Tobramycin(10 μg)	37 (41.1)	8(8.8)	45 (50)

### PCR and sequencing

**Fig 1:**
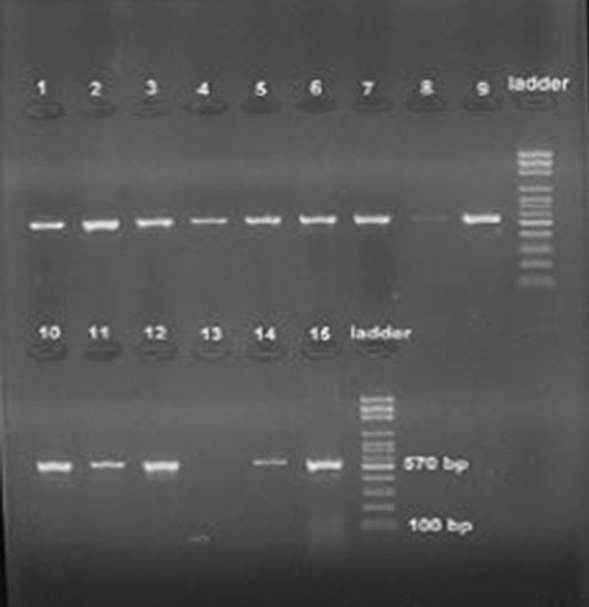
Agarose gel electrophoresis for amplification analysis of *oprD* Gene. Lane 1-12,14,15: *oprD* PCR result, lane 13: negative control, DNA ladder: 50 bp. The size of the amplified fragment is 570 base pairs

The *oprD* gene and genomic region upstream of *oprD* were amplified by PCR. The electrophoresis agarose gel was performing on PCR products that were shown in Fig. 1. The size of the amplified fragment is 570 base pairs. As shown in Fig. 1.

The *oprD* gene was sequenced, including the promoter and upstream regions including Shine-Dalgarno (GGAG; nucleotides − 12 to − 9), − 10 (TAAGTT; nucleotides − 84 to − 79), and − 35 (TCGCCA; nucleotides − 107 to − 102) sequences. Of 29 isolates selected for sequencing, 25 (86.2%) of isolates had mutations that all (100%) the isoltes were resistant to imipenem and four (13.7%) isolates had no mutations. Mutations’ percentages in resistant and isolates are shown in Table 2. There was a significant relationship (P < 0.05) between mutations upstream of the *oprD* coding regions and MIC of imipenem.

**Table 2:** The relation between mutation and MIC

E-test	Mutation		Total
	yes	No	
Resistant	22 (95.6%)	1(14.2%)	23 (76.6%)
Sensitive	1 (4.3%)	6 (85.7%)	7 (23.3%)
Total	23(100%)	7 (100%)	30 (100%)

The frequency of mutations based on specimens was as follows: Burn 57.69%, Urine 19.23%, and other specimens 23.07%. Most mutations were seen in *P. aeruginosa* isolated from burn specimens and burn ward. The Statistical analysis found a significant correlation between the type of specimens and MIC (P ≤ 0.05). The Statistical analysis found a significant correlation between MIC and resistance to imipenem (P ≤ 0.05).

Based on the observed mutations, none of the strains had no mutation in Shine-Dalgarno (GGAG; nucleotides − 12 to − 9), − 10 (TAAGTT; nucleotides − 84 to − 79), and − 35 (TCGCCA; nucleotides − 107 to − 102) sequences. Six isolates have point mutations in the promoter, Five isolates had T→C base substitution at nt -90 and One isolate had a base substitution G→ Cat nt − 120. Also, One isolate had a base substitution A→ Cat n t 25 in the coding region, and this isolate had a point mutation leading to an amino acid change at positions 9(I→L). The insertion of one base was seen in five isolates and the insertion of tree nucleotide was observed in one isolate. The rest of the results sequencing of upstream regions and promoter regions are shown in Table 3.

**Table 3:** The result of sequencing

**N** NO. of isolate	Description of mutation	Resistant/sensitive
115	b→ T base substitution at nt 353 G→T base substitution at nt 437 C→T base substitution at nt 437 T→C base substitution at nt 443	R
18	G→A base substitution at nt 436	R
125	A→C base substitution at nt 464	R
49	G→T base substitution at nt 486	R
57,58,16,56,55,10	T→C base substitution at nt 648	R: 57,58,55,10,56 S: 16
53,78,68,126,125	C→T base substitution at nt 452 C→T base substitution at nt 596	R
200,202,203,31,53,68,80,115,125,126	T→A base substitution at nt 296 A→ G base substitution at nt 308 A→ G base substitution at nt 313 C→T base substitution at nt 340 A→ G base substitution at nt 381 G →A base substitution at nt 467 G →A base substitution at nt 572 G →A base substitution at nt 593 A→G base substitution at nt 595	R
200,202,203	Deletion of 2 bp (AC) at nt 529-530	R
48,49,17,122,18,203	G→A base substitution at nt 481	R
17,49,55,56,10,122	Insertion of 1 bp (C) at nt 299	R

## Discussion

In medicine, the treatment of community-acquired infections and nosocomial infections caused by *P. aeruginosa is* important. Carbapenem is effective against infectious diseases caused by P. aeruginosa. However, carbapenem-resistant *P. aeruginosa* strains are emerging worldwide, and the rate of resistance in most countries ranges from 10 to 50 % (Huang, Jeanteur, Pattus, & Hancock, 1995). In the present study, the prevalence of imipenem resistance in bacteremic *P. aeruginosa* was 48.9 %, and the rate of resistance of *P. aeruginosa* to imipenem was 5.5 % to 62.5% in other studies(Dantas et al. 2017; Dubois et al. 2008; Gill et al. 2011; Hammami et al. 2009; Kohanteb et al. 2007; Lei et al. 2003; Levine et al. 1998; Sapino et al. 2012; Zarei-Yazdeli et al. 2014).

According to studies of antibiotic resistance in different parts of the world and the result of the present study; it can be concluded that resistance rates in *P. aeruginosa* isolates were higher than previous reports, which can be due to a combination of different factors such as the inconsiderate use or the previous use of antibiotics in prophylaxis, differences in the type of sample, and the geographical region and care of patients in hospitals and difference in the mechanism of resistance‏. Since the carbapenems are commonly used in the treatment and mutations in the genomic region upstream of *oprD* and promoter are the most current reason against resistance to these antibiotics, so identifying and assessing the prevalence of these mutations in the bacteria population can be very effective in controlling the resistance pattern. The mutational inactivation of the *oprD* gene and disruption in promoter represents the major cause of *OprD* loss in *P. aeruginosa* strains. In our study, alterations were observed in resistant isolates. Mutations of the upstream region *oprD* gene were seen in all (25) the imipenem-resistant isolates. Mutations in SS1 and SS2 were point mutations. One isolate had a base substitution A→ Cat n t 25 in the coding region and this isolate had a point mutation leading to an amino acid change at positions 9 (I→L). Also, the insertion of one base was seen in five isolates and the insertion of tree nucleotide were observed in one isolate.

A similar study was performed by Damien Fournier et al (Fournier et al. 2013). Mutations of the *oprD* gene were seen in 86.2% of imipenem-resistant isolates and Reported the lack of *OprD* was due to tot the disruption of the *oprD* promoter by ISPsy2 in one strain and the other strains had a mutation or gene disruption by different insertion sequences ISPa1635, ISPa1328, IS911, ISPs1, IS51, IS222, and ISPa41). In a study conducted by Alain A et al (Ocampo-Sosa et al. 2012). seventy-seven (77%) isolates had mutations and mutations were observed in both sensitive and resistant isolates. Most isolates showed point mutations and deletion mutations. In a study performed by Aki Hirabayashi et al (2017). Sequencing of *oprD* gene and the promoter and downstream regions were done and the results revealed that most of the resistant-isolates had insertion mutations in the *oprD* gene, also there was a direct relationship between the alteration or loss of *oprD* and the increase in MIC, for imipenem but not meropenem and other carbapenems (Cowan et al. 1992; Ocampo-Sosa et al. 2012; Shen et al. 2015; Zarei-Yazdeli et al. 2014). ‏In a study conducted by Yumiko Sanbongi et al (2009). Most mutations were frame-shift mutations or deletion mutations. Gutiérrez et al (2007). Have reported different mutations in the *oprD* gene, the most frequent mutations were frameshift mutations produced by one nucleotide insertions or deletions and point mutations leading to the creation of a premature stop. In a study performed by EL Amin et al (2005). Sequence analysis revealed mutation of inactivation, including the insertion or deletion of one and two or more nucleotides and insertion sequences (IS). In investigating Performed by Wolter DJ et al (2004). PCR and sequence analysis revealed an interpolation of a large fragment in the *oprD* gene was known as IS elements that are not observed in this study. Jill Shen et al (2015). Reported 96.5% (136/141) of the resistant isolates had mutations. Ninety-six strains had a small deletion in the *OprD* gene or multi-site mutations and 34 strains had a large deletion in the *OprD* gene, 6 strains had IS, and 4 strains had no mutation and showed a normal *OprD2* gene. In this study, the insertion of one base was seen in five strains. Twenty-five strains had point mutations and 4 strains had no mutation.

Yoneyama et al (1993). Reported a large deletion encompassing a region from upstream to downstream across the promoter region (from nucleotides 519-685) that prevented transcription of *oprD* and also deletion mutations were observed, including deletion an11 bp. Qinghui Sun et al (2016). Have reported an insertion sequence element (ISRP10) that causes disrupt of the *oprD* gene and is seen in 96% of imipenem-resistant *P. aeruginosa* isolates. In a study performed by Yingjun Yan et al (2014). The result of the analysis, indicatied that the 4-bp insertion in the *oprD* gene resulted in a frameshift in the *OprD* gene and imipenem resistance.

A different study conducted by Hussein Chalhoub (2016). DNA sequencing showed several mutations in the coding region *oprD*, but no mutation was observed in the promoter region of the gene. Reports had shown that mutation and inactivation or loss of an *oprD* gene, disruption in promoter and upstream region of *oprD* gene in *P. aeruginosa* strains are the major mechanisms that cause resistance to imipenem‏. This result was in accordance with the previous investigation of the clinical isolates of *P. aeruginosa*.

The results of this study show, increase in the resistance of *P. aeruginosa* to imipenem. Sequencing of the genomic region upstream of *oprD* in clinical strains revealed the point mutations in resistant strains. One isolate had a base substitution in the coding region *oprD* gene and this isolate had a point mutation leading to an amino acid change. All the imipenem-resistant isolates had mutations and Sensitive strains had no mutation. Judicious use of antimicrobials and controlled usage of imipenem may prevent *P. aeruginosa* from acquiring resistance to IPM. Neutralization of the mutation or the presence of a substance that can inactivate the mutation could lead to bacterial susceptibility to imipenem antibiotics.In our country, there is little information about the contribution of different mechanisms to imipenem resistance in these isolates, especially about *oprD* mutations in the upstream region of gene and promoter in imipenem-resistance isolates. Awareness of resistant mechanisms in *P.aeruginosa* isolates could help to regulate infection control strategies and to enhance the efficacy of imipenem for the treatment of infections due to these bacteria. Thus, there is a need to focus on intrinsic resistance mechanisms, especially Porin alteration which also confers significant imipenem resistance, it also suggests in the future other mechanisms such as gene expression and its relationship with the *oprD* mutations are evaluated and investigated in other isolates and other places.

## Data Availability

The data are available. All data generated or analyzed during this study are included in this study.

## References

[CR1] Amin NE, Giske CG, Jalal S, Keijser B, Kronvall G, Wretlind B (2005). Carbapenem resistance mechanisms in *Pseudomonas aeruginosa*: alterations of porin *OprD* and efflux proteins do not fully explain resistance patterns observed in clinical isolates. Apmis.

[CR2] Chalhoub H, Sáenz Y, Rodriguez-Villalobos H, Denis O, Kahl BC, Tulkens PM, Van Bambeke F (2016). High-level resistance to meropenem in clinical isolates of *Pseudomonas aeruginosa* in the absence of carbapenemases: role of active efflux and porin alterations. Int J Antimicrob Agents.

[CR3] Cowan S, Schirmer T, Rummel G, Steiert M, Ghosh R, Pauptit R, Jansonius JN, Rosenbusch J (1992). Crystal structures explain functional properties of two E. coli porins. Nature.

[CR4] Dantas RC, Silva RT, Ferreira ML, Gonçalves IR, Araújo BF, Campos PA, Royer R, William D, Batistão F, Gontijo-Filho P, Ribas RM (2017). Molecular epidemiological survey of bacteremia by multidrug resistant *Pseudomonas aeruginosa*: the relevance of intrinsic resistance mechanisms. Plos One.

[CR5] Dubois V, Arpin C, Dupart V, Scavelli A, Coulange L, André C, Fischer I, Grobost F, Brochet J, Dutilh B, Jullin J, Noury P, Larribet G, Quentin C (2008). β-Lactam and aminoglycoside resistance rates and mechanisms among *Pseudomonas aeruginosa* in French general practice (community and private healthcare centres). J Antimicrob Chemother.

[CR6] Fournier D, Richardot C, Müller E, Robert-Nicoud M, Llanes C, Plésiat P, Jeannot K (2013). Complexity of resistance mechanisms to imipenem in intensive care unit strains of *Pseudomonas aeruginosa*. J Antimicrob Chemother.

[CR7] Gill MM, Usman J, Kaleem F, Hassan A, Khalid A, Anjum R, Fahim Q (2011). Frequency and antibiogram of multi-drug resistant *Pseudomonas aeruginosa*. J Coll Physicians Surg Pak.

[CR8] Gutiérrez O, Juan C, Cercenado E, Navarro F, Bouza E, Coll P, J L, Pérez. Oliver, A. (2007). Molecular epidemiology and mechanisms of carbapenem resistance in *Pseudomonas aeruginosa* isolates from Spanish hospitals. Antimicrob Agents Chemother.

[CR9] Hammami S, Ghozzi R, Burghoffer B, Arlet G, Redjeb S (2009). Mechanisms of carbapenem resistance in non-metallo-β-lactamase-producing clinical isolates of *Pseudomonas aeruginosa* from a Tunisian hospital. Pathologie Biologie.

[CR10] Hancock RE, Brinkman FS (2002). Function of *Pseudomonas* porins in uptake and efflux. Ann Rev Microbiol.

[CR11] Hirabayashi A, Kato D, Tomita Y, Iguchi M, Yamada K, Kouyama Y, Morioka H, Tetsuka N, Yagi T (2017). Risk factors for and role of *OprD* protein in increasing minimal inhibitory concentrations of carbapenems in clinical isolates of *Pseudomonas aeruginosa*. J Med Microbiol.

[CR12] Huang H, Jeanteur D, Pattus F, Hancock RE (1995). Membrane topology and site-specific mutagenesis of *Pseudomonas aeruginosa* porin *OprD*. Mol Microbiol.

[CR13] Kohanteb J, Dayaghi M, Motazedian M, Ghayumi M-A (2007). Comparison of biotyping and antibiotyping of *Pseudomonas aeruginosa* isolated from patients with burn wound infection and nosocomial pneumonia in Shiraz Iran. PJBS.

[CR14] Lei Y, Wang H, Sun Z, Shen Z (2003). Susceptibility of 570 *Pseudomonas aeruginosa* strains to 11 antimicrobial agents and the mechanism of its resistance to fluoroquinolones. Zhonghua yi xue za zhi.

[CR15] Levine C, Hiasa H, Marians KJ (1998). DNA gyrase and topoisomerase IV: biochemical activities, physiological roles during chromosome replication, and drug sensitivities. Biochimica et Biophysica Acta.

[CR16] Lister PD, Wolter DJ, Hanson ND (2009). Antibacterial-resistant *Pseudomonas aeruginosa*: clinical impact and complex regulation of chromosomally encoded resistance mechanisms. Clin Microbiol Rev.

[CR17] Lynch M, Drusano G, Mobley H (1987). Emergence of resistance to imipenem in *Pseudomonas aeruginosa*. Antimicrob Agents Chemother.

[CR18] Ocampo-Sosa AA, Cabot G, Rodríguez C, Roman E, Tubau F, Macia MD, Zamorano Moya B, L, Suárez C, Peña, C. (2012). Alterations of *OprD* in carbapenem-intermediate and-susceptible strains of *Pseudomonas aeruginosa* isolated from bacteremia in a Spanish multicenter study. Antimicrob Agents Chemother.

[CR19] Pirnay JP, Vos DD, Mossialos D, Vanderkelen A, Cornelis P, Zizi M (2002). Analysis of the *Pseudomonas aeruginosa oprD* gene from clinical and environmental isolates. Environ Microbiol.

[CR20] Riera E, Cabot G, Mulet X, García-Castillo M, del Campo R, Juan R, Cantón. Oliver, A. (2011). *Pseudomonas aeruginosa* carbapenem resistance mechanisms in Spain: impact on the activity of imipenem, meropenem and doripenem. J Antimicrob Chemother.

[CR21] Rostami S, Sheikh AF, Shoja S, Farahani A, Tabatabaiefar MA, Jolodar A, Sheikhi R (2018). Investigating of four main carbapenem-resistance mechanisms in high-level carbapenem resistant *Pseudomonas aeruginosa* isolated from burn patients. J Chin Med Assoc.

[CR22] Sanbongi Y, Shimizu A, Suzuki T, Nagaso H, Ida T, Maebashi K, Gotoh N (2009). Classification of *OprD* sequence and correlation with antimicrobial activity of carbapenem agents in *Pseudomonas aeruginosa* clinical isolates collected in Japan. Microbiol Immunol.

[CR23] Sapino B, Mazzucato S, Solinas M, Gion M, Grandesso S (2012). Comparison of different methods for determining beta-lactam susceptibility in *Pseudomonas aeruginosa*. Microbiologica.

[CR24] Shen J, Pan Y, Fang Y (2015). Role of the outer membrane protein *OprD2* in carbapenem-resistance mechanisms of *Pseudomonas aeruginosa*. Plos One.

[CR25] Sun Q, Ba Z, Wu G, Wang W, Lin S, Yang H (2016). Insertion sequence ISRP10 inactivation of the *oprD* gene in imipenem-resistant *Pseudomonas aeruginosa* clinical isolates. Int Antimicrob Agents.

[CR26] Wolter DJ, Hanson ND, Lister PD (2004). Insertional inactivation of *oprD* in clinical isolates of *Pseudomonas aeruginosa* leading to carbapenem resistance. FEMS Microbiol Lett.

[CR27] Yan Y, Yao X, Li H, Zhou Z, Huang W, Stratton CW, Lu C-D, Tang Y-W (2014). A novel *oprD*-mutated *Pseudomonas aeruginosa* strain in relation to a nosocomial respiratory infection outbreak in an intensive care unit. J Clin Microbiol.

[CR28] Yoneyama H, Nakae T (1993). Mechanism of efficient elimination of protein *D2* in outer membrane of imipenem-resistant *Pseudomonas aeruginosa*. Antimicrob Agents Chemother.

[CR29] Zarei-Yazdeli M, Eslami G, Zandi H, Mousavi SM, Kosha H, Akhavan F, Kiani M (2014). Relationship between antimicrobial resistance and class I integron in Pseudomonas aeruginosa isolated from clinical specimens in Yazd during 2012–2013. Feyz J Kashan Univ Med Sci.

